# Comparing classification methods for diffuse reflectance spectra to improve tissue specific laser surgery

**DOI:** 10.1186/1471-2288-14-91

**Published:** 2014-07-16

**Authors:** Alexander Engelhardt, Rajesh Kanawade, Christian Knipfer, Matthias Schmid, Florian Stelzle, Werner Adler

**Affiliations:** 1 Department of Medical Informatics, Biometry and Epidemiology, Friedrich-Alexander University Erlangen-Nuremberg, Waldstrasse 6, 91054 Erlangen, Germany; 2 Department of Oral and Maxillofacial Surgery, Erlangen University Hospital, Glückstrasse 11, 91054 Erlangen, Germany; 3 Department of Medical Biometry, Informatics and Epidemiology, University of Bonn, Sigmund-Freud-Strasse 25, 53105 Bonn, Germany; 4 SAOT - Graduate School in Advanced Optical Technologies, Paul-Gordan-Strasse 6, 91052 Erlangen, Germany

**Keywords:** Laser surgery, Reflectance spectroscopy, Machine learning, Penalized discriminant analysis

## Abstract

**Background:**

In the field of oral and maxillofacial surgery, newly developed laser scalpels have multiple advantages over traditional metal scalpels. However, they lack haptic feedback. This is dangerous near e.g. nerve tissue, which has to be preserved during surgery. One solution to this problem is to train an algorithm that analyzes the reflected light spectra during surgery and can classify these spectra into different tissue types, in order to ultimately send a warning or temporarily switch off the laser when critical tissue is about to be ablated. Various machine learning algorithms are available for this task, but a detailed analysis is needed to assess the most appropriate algorithm.

**Methods:**

In this study, a small data set is used to simulate many larger data sets according to a multivariate Gaussian distribution. Various machine learning algorithms are then trained and evaluated on these data sets. The algorithms’ performance is subsequently evaluated and compared by averaged confusion matrices and ultimately by boxplots of misclassification rates. The results are validated on the smaller, experimental data set.

**Results:**

Most classifiers have a median misclassification rate below 0.25 in the simulated data. The most notable performance was observed for the Penalized Discriminant Analysis, with a misclassifiaction rate of 0.00 in the simulated data, and an average misclassification rate of 0.02 in a 10-fold cross validation on the original data.

**Conclusion:**

The results suggest a Penalized Discriminant Analysis is the most promising approach, most probably because it considers the functional, correlated nature of the reflectance spectra.

The results of this study improve the accuracy of real-time tissue discrimination and are an essential step towards improving the safety of oral laser surgery.

## Background

Oral and maxillofacial surgery is a field where precise cuts with minimal operative trauma are of particular interest. Laser scalpels are able to operate much more precisely than metal scalpels. The development of laser surgery has brought forward a new method of performing surgery which reduces the risk of infection that arises when introducing a metal scalpel in the patient’s body. By using a laser scalpel instead, this source of infection risk is nearly eliminated, because no foreign body is inserted into the patient’s tissue. Postoperative healing time has been shown to decrease as well [1]. However, this technology also introduces a new problem: When using a traditional metal scalpel, surgeons can feel if they are about to cut a different type of tissue such as, a nerve—this is called *haptic feedback*—and are thus able to work around that crucial tissue. Preserving this tissue is of utmost importance, as irreparable nerve damage is one of the most serious side-effects of surgery. Especially in oral and maxillofacial surgery, situations arise where the complex anatomy in the head and neck region predestinates risks for certain kinds of surgeries. As an example, removing the parotid gland can be a dangerous procedure as branches of the facial nerve run directly through the gland [2].

Laser scalpels, however, cannot provide haptic feedback. For this reason, it is necessary to develop alternative methods to detect what type of tissue (e.g. fat, nerve, blood vessels) is being operated on during a surgery. One possible way, applied in this study, is to illuminate the region at and near the scalpel’s current position, and to measure the reflected light spectra. These spectra are given as smooth real-valued functions over a certain wavelength range, and are measured at discrete wavelenghts. Data of this type is called *functional data*[3]. When these spectra are measured in between pulses of the laser scalpel, one can then classify them and find out what tissue is about to be ablated. Finally, this can then be assembled into an algorithm that detects when the laser is about to damage critical tissue and emits a warning signal to the surgeon or temporarily shuts off the laser as an automatic feedback mechanism.

### Spectroscopy

For the purpose of tissue diagnostics, two popular methods of spectroscopy, diffuse reflectance spectroscopy and autofluorescence spectroscopy, are available [4,5]. The most widely used method uses *autofluorescence spectroscopy* to derive a tissue’s characteristics.

When a tissue sample is illuminated with light of specific wavelengths, certain molecules called fluorophores (including amino acids, vitamins and lipids) absorb the light’s energy and subsequently emit light themselves, this time of lower energy, i.e. longer wavelengths. The concentration of some fluorophores changes in different tissue types, thus altering the resulting autofluorescence spectra and enabling tissue discrimination.

This study makes use of *diffuse reflectance spectroscopy*, which exploits the differences in absorption and scattering properties of various tissue types. The tissue is illuminated with white light, and a spectrometer then measures the degree of single and multiple backscattering.

The question which method is preferrable has no concrete answer as of now. In a breast cancer detection study, Breslin et al. [5] found that autofluorescence spectroscopy is the superior method for discriminating benign from malignant breast tissue. De Veld et al. [4] however found that the analysis of diffuse reflectance spectra and autofluorescence spectra performed equally well when differentiating oral lesions from healthy tissue, but diffuse reflectance spectra were better for discriminating benign from malignant lesions.

One might conclude that the two methods perform similarly in cancer detection tasks. However, Douplik et al. [6] showed that autofluorescence spectra are altered under laser ablation conditions, suggesting that another method of spectroscopy might be more viable in a laser surgery setting.

### Existing work

Previous, related studies [2,7-9] that developed a tissue detection algorithm compared the total misclassification error, i.e. the ratio of falsely classified observations to all observations, as well as the pairwise ROC curves [10], which plot the false positive rate ($\frac{\mathrm{false}\;\text{positives}}{\mathrm{false}\;\text{positives}+\mathrm{true}\;\text{negatives}}$, equal to 1 minus the specificity and ranging from 0 to 1) on the *x*-axis vs. the true positive rate ($\frac{\mathrm{true}\;\text{positives}}{\mathrm{false}\;\text{negatives}+\mathrm{true}\;\text{positives}}$, equal to the sensitivity and ranging from 0 to 1) on the *y*-axis. Because an ROC curve an only compare two tissue types at a time, one has to use pairwise curves, one for each tissue pair. Lastly, the pairwise area under the ROC curve (AUC), which ranges from 0.5 for random guessing to 1.0 for a perfect classification, was compared.

Stelzle et al. [2,7] used ex-vivo pig heads to measure and classify the diffuse reflectance spectra of hard and soft tissue types, namely skin, muscle, mucosa, subcutaneous fat, salivary gland, bone, and nerve tissue. They found that almost all tissue pairs could be differentiated with high sensitivity and specificity. Many tissue pairs could be differentiated with sensitivities and specificities of around 90%. Some sensitivities and specificities were slightly lower, but the results still show the feasibility of remote tissue differentiation. One of the problems that persist in these two studies is that measurements were taken ex-vivo, i.e. from dead tissue. The fact that blood flow to this tissue had stopped before measurements were taken implies that measurements of in-vivo tissue will differ slightly, depending on the additional light absorption and scattering properties of blood occuring in live tissue.

In a follow-up study, Stelzle et al. [8] took on the above mentioned problem of ex-vivo tissue alterations. The authors classified the diffuse reflectance spectra of four soft tissue types (skin, fat, muscle, nerve) of live rats. This study achieved an AUC and a sensitivity as well as specificity of 1.00 for all tissue pairs except skin/fat. These results may suggest that, due to the additional occurence of blood in live tissue, classification performance is improved under in-vivo conditions.

Most recently, Stelzle et al. [9] classified the diffuse reflectance spectra of five tissue types of ex-vivo pig heads. This study differed in the fact that measurements were taken before and after the ablation of tissue with a laser scalpel, comparing the results and drawing conclusions about the effects of laser ablation on prediction strength. The study found that after laser ablation, the total classification error rose from around 14% to 17%. Only the differentiation between the tissue types nerve/fat was enhanced under laser ablation conditions.

All of the mentioned studies reach high sensitivities and specificities, concluding that a tissue differentiation by diffuse reflectance spectroscopy is feasible, even in live tissue and under laser ablation conditions.

However, all of the previously listed work used the same method of analysis: The dimension of the measured spectra was reduced by a Principal Component Analysis (PCA) [11], and the PC scores were subsequently classified by a Linear Discriminant Analysis (LDA) [12].

Moreover, the data in the four above-mentioned cases came from a relatively small number of specimen, where each animal has been measured many times. This resulted in a large sample size, but highly correlated spectra within animals, due to the repeated measurements.

### Purpose of this study

The objective of this study is to extend the existing work in two ways: Firstly, the problem of the repeated, correlated measurements is tackled by averaging out the repeated spectra within each animal. Because the variance of the spectra within each animal is much smaller than the variance between animals, this procedure reduces the amount of data without reducing the amount of information much, and furthermore removes a possible downward bias when estimating the covariance matrices for each tissue type. Afterwards, new spectra are simulated by estimating mean vectors and covariance matrices for each tissue class separately, and using these estimates to simulate new spectra according to multivariate Gaussian distributions. Secondly, the study’s main objective is to apply a series of classification algorithms to the simulated data, and find out if other classification methods have a better prediction strength than the previously applied LDA.

The insights acquired from this study can be used in further research and suggest a set of algorithms to concentrate on for subsequent studies.

## Methods

### Data acquisition and preprocessing

#### *Data acquisition*

The data consist of diffuse reflectance spectra measured at a discrete set of wavelengths, taken from different tissue types of a set of ex-vivo pig heads. The initial data set used in this study is a merged data frame, combined from the data sets used in Stelzle et al. [2,7].

Measurements were taken from twelve dissected pig heads obtained from a slaughterhouse. For each pig head, eight tissue types were dissected, from each of which six spots were selected to take reflectance spectra from. Finally, 30 measurements per spot were made. The many repetitions were taken because once a tissue sample is readily lying under the spectrometer, additional measurements are done quickly and cheaply.

Thus, in total, the complete data consisted of 12 animals · 8 tissue types · 6 spots · 30 repetitions = 17280 diffuse reflectance spectra. The spectra were measured at 1150 discrete wavelengths between 350 nm and 650 nm, in steps of around 0.26 nm. The tissues were illuminated with a pulsed Xenon lamp, and the reflected spectra were measured with a backscattering probe that transferred the measurements to a spectrometer. Refer to Stelzle et al. [7] for further details on the experimental set up.

#### *Preprocessing*

To bring the data into a structure that is in agreement with the prerequisites for simulation, the data was thinned out by averaging over the repetitions and spots. This procedure was justified by looking at 30 measurements of each spot and tissue type, which showed only minimal variation, and 6 measurements of each animal and tissue type at its different spots, which show almost exclusively only a constant vertical shift.

The averaging was done first and foremost to eliminate redundant information in the data. Secondly, it was believed that keeping the repetitions would result in a too small estimate for the covariance matrices within each tissue class, because the data essentially contained 30 copies of each spectrum. By making sure that each spectrum in the final data set belongs to a different animal, it can be assumed that the subsequent simulation of new spectra generates spectra of new animals instead of new spots.

**Figure 1:**
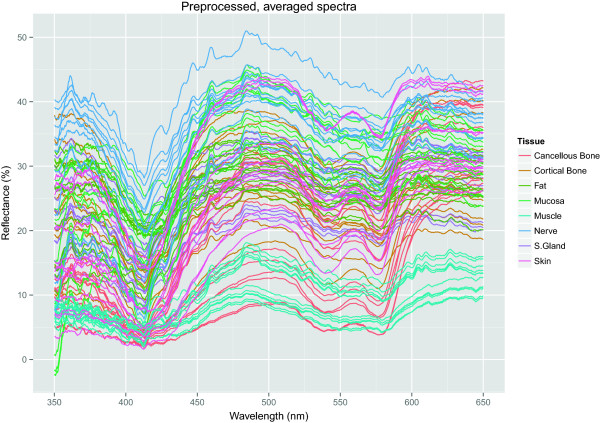
**All 96 spectra, 8 tissue types for each of the 12 animals, after preprocessing has taken place.** This is the data set with which the simulation has been carried out. Each of the spectra is actually an average over six spots and 30 repetitions, i.e. 180 of the spectra of the original data set have been averaged into one line of this figure.

The preprocessed data set contained 96 observations (8 tissue types · 12 animals) of 1154 columns, described in Table 1 and shown in Figure 1.

**Table 1:** A description of the columns in the data

**Variable name**	**Description**
350.14	Reflectance measured at the wavelength of
	350.14 nm. A floating point number typically in the
	range from 0 to 60.
350.41	Reflectance measured at the wavelength of 350.41 nm
⋮	⋮
649.98	Reflectance measured at the wavelength of 649.98 nm
Specimen	The ID of the animal measured (integer between 1
	and 12).
Tissue	Which tissue type was measured (categorical
	variable, possible values are Fat, Mucosa, Muscle,
	Nerve, Skin, Cortical Bone, Salivary Gland,
	Cancellous Bone)

### Simulation of new spectra

Since the previous studies were carried out with many repeated measurements of a small number of specimen, many of the measurements were highly correlated, and the authors remarked that the sample size was actually rather small, considering the degree of redundancy in the data set.

For that reason, the data set was first shrunk by averaging out the repetitions, and subsequently enlarged via simulation. The simulation created spectra that can be interpreted as measurements from *new* animals rather than additional repetitions of the same animal.

After preprocessing, the remaining data set consisted of *N* = 96 spectra, each belonging to one of *C* = 8 tissue types. The number of spectra in each of the *C* tissue classes is denoted by *n*_*c*_, *c*∈1,…,*C*, and is constant in this data set, *n*_*c*_ = 12 ∀ *c*.

#### *Simulation procedure*

The simulation was conducted by using the following procedure:

First, split the data frame of the spectra (denoted by ***X***) into *C* = 8 subsets, one for each tissue type *c*. Denote the resulting matrices of reflectance spectra by $X_{c}\in R^{n_{c}\times p}$. Assume in each of the *C* tissue classes *c*∈1,…,*C*, each one of the *n*_*c*_ spectra follows a *p*-dimensional multivariate normal distribution with mean *μ*_*c*_ and covariance matrix *Σ*_*c*_,

$$x_{j}∼N_{p}(\mu_{c},\Sigma_{c}),\;j=1,\ldots ,n_{c}.$$

One can then obtain simple parameter estimators ${\hat{\mu }}_{c}$ and ${\hat{\Sigma }}_{c}$ for every class *c* separately:

(1) $$\begin{array}{rr} {\hat{\mu }}_{c} & =\frac{1}{n_{c}}\left(\begin{array}{r} \overset{n_{c}}{\underset{i=1}{\sum }}x_{i1}\\ \;\vdots \\ \overset{n_{c}}{\underset{i=1}{\sum }}x_{\text{ip}} \end{array}\right)\; \end{array}$$

(2) $$\begin{array}{rr} {\hat{\Sigma }}_{c} & =\frac{1}{n_{c}-1}X_{c}^{⊤}X_{c}\; \end{array}$$

We can subsequently use the obtained estimators to simulate *m*_*c*_ additional observations in each class *c*:

$$x_{j}^{*}∼N_{p}({\hat{\mu }}_{c},{\hat{\Sigma }}_{c})\;,\;j=1,\ldots ,m_{c},$$

where ${\hat{\mu }}_{c}$ and ${\hat{\Sigma }}_{c}$ are the respective estimates for *μ*_*c*_ and *Σ*_*c*_.

#### *The estimation data sets*

**Figure 2:**
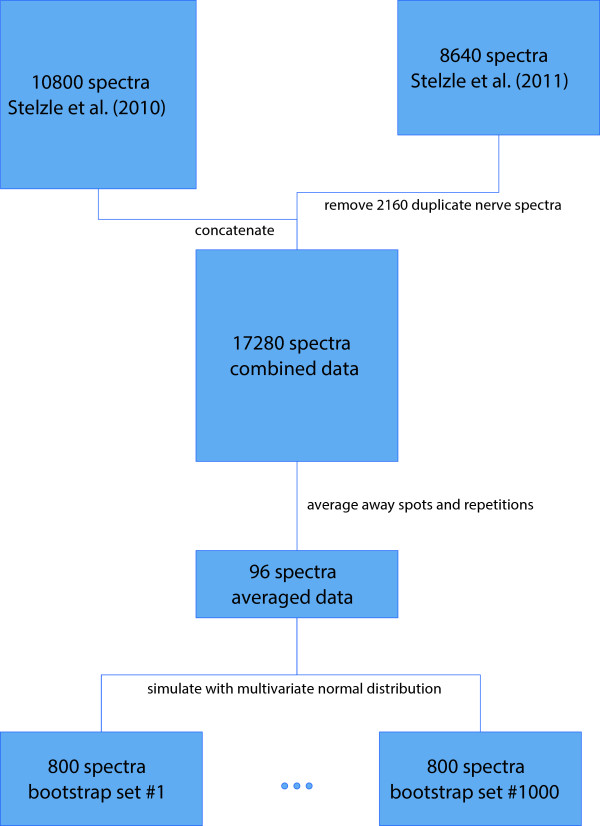
A flowchart that summarizes how the data was manipulated.

Using the estimated means $\hat{\mu_{c}}$ and covariances ${\hat{\Sigma }}_{c}$, new data was simulated to obtain a distribution of error rates. We generated 1000 data sets of 800 observations (*m*_*c*_ = 100) each. These data sets were used to train classification algorithms and generate boxplots of their misclassification rates, and to compute confusion matrices to allow for more detailed insights in where misclassification occurs.Figure 2 summarizes the data manipulation process. For the subsequent analyses, the original 96 spectra were neglected for simplicity; only simulated spectra were considered.

### Dimension reduction with Principal Component Analysis (PCA)

Because the measured spectra consist of many highly correlated input variables which can lead to instable parameter estimates, a PCA [11] was applied as a method of dimensionality reduction.

To allow a comparison between using a PCA and using the original spectra, the classification algorithms were each carried out first with the original spectra, and subsequently with only a number of PC scores.

The computation of the principal components relies heavily on the (sample) covariance matrix ***Σ*** of the data ***X***, which is the row-wise matrix composed of the *n* observations ***x***_*i*_. The *k*-th principal component is given by $z_{k}=\alpha_{k}^{⊤}x$, where ***α***_*k*_ is the eigenvector of ***Σ*** corresponding to the *k*-th largest eigenvalue *λ*_*k*_ of ***Σ***.

The idea of a PCA is to use a small number *q*≪*p* of principal components that still explain a sufficient proportion of the total variance, thus reducing the number of input variables while losing only a minimal amount of available information.

To choose the number *q* of principal components for further analyses, a multitude of methods is available. In this study, the *average eigenvalue criterion* was applied because it is an objective, computationally low-cost method which has shown good results according to Valle et al. [13]. With the average eigenvalue criterion, one selects all PCs with an associated eigenvalue greater than the average eigenvalue of all PCs.

### Classification methods

The simulation and analyses were carried out with the open programming environment R, version 3.0.0 [14]. The following additional packages were used: 

• For linear discriminant analysis (LDA) and quadratic discriminant analysis (QDA): The MASS package [15].

• For penalized discriminant analysis (PDA): The mda package [16].

• For Random Forests: The randomForest package [17].

• For classification and regression trees (CART): The rpart package [18].

• For Neural Networks: The nnet package [15].

• For *k*-nearest-neighbor: The class package [15].

• For the graphics: The reshape and ggplot2 packages [19,20].

Where possible, each classification algorithm was executed with both the original spectra as well as the PC scores. For some classifiers, one of the methods was either impossible or nonsensical: 

• For LDA and QDA, highly correlated covariates (as is the case for discretized functions) lead to unstable estimates. For that reason, only PC scores were analyzed with LDA and QDA.

• PDA has its strength in the case of many correlated covariates. It thus makes little sense to analyze a set of PC scores with that algorithm, which is why only the spectra were considered for PDA.

The classification algorithms used in this study are described only briefly here. Some of the algorithms can be run out-of-the-box, whereas others depend on some *tuning parameters* or *hyperparameters*, which influence the course of action of the algorithm. Where necessary, these tuning parameters are described, as well. Refer to Hastie et al. [12] for an extensive description of all algorithms discussed here.

#### *k-nearest-neighbor*

A *k*-nearest-neighbor classifier [21] takes a new observation vector *x*^∗^, and finds its *k* nearest neighbors in the training sample. The definition of *closest* point is subject to some chosen distance metric, most commonly the euclidean distance, which is defined as $d(u,v)=\sqrt{\overset{k}{\underset{l=1}{\sum }}{\left(u_{l}-v_{l}\right)}^{2}}$ for two vectors *u* and *v*. The predicted class for *x*^∗^ is then the majority vote of the response variable of the nearest neighbors.

The *k*-nearest-neighbor algorithm has only one hyperparameter, namely *k*.

#### *Linear discriminant analysis (LDA)*

Linear discriminant analysis [22] is based on imposing a multivariate Gaussian mixture model upon the training data i.e. it assumes: 

• A class probability *π*_*c*_ for each class *c*, subject to $\overset{C}{\underset{c=1}{\sum }}\pi_{c}=1$.

• In each class *c* the input data *x*_*i*_ follow a multivarate Gaussian distribution:

$$\begin{array}{l} f(x|Y=c)=\frac{1}{{\left(2\pi \right)}^{p/2}{\left|\Sigma_{c}\right|}^{1/2}}\\ \;\exp\left(-\frac{1}{2}{\left(x-\mu_{c}\right)}^{⊤}\Sigma_{c}^{-1}(x-\mu_{c})\right). \end{array}$$

• Linear (as opposed to quadratic) discriminant analysis assumes the covariance matrices within all classes are equal, i.e. ***Σ***_*c*_ = ***Σ*** ∀ *c*.

After estimating the *π*_*c*_, *μ*_*c*_ and ***Σ*** from the training data set , the decision rule can be described with a set of *discriminant functions*

$$\delta_{c}(x^{*})={\left(x^{*}\right)}^{⊤}\Sigma^{-1}\mu_{c}-\frac{1}{2}\mu_{c}^{⊤}\Sigma^{-1}\mu_{c}+\log\left(\pi_{c}\right).$$

LDA owes its name to the fact that these discriminant functions are linear in *x*^∗^. The fitting function is then $Ŷ(x^{*})={\text{argmax}}_{c}\;\delta_{c}(x^{*})$.

#### *Quadratic discriminant analysis (QDA)*

In some situations (the simulation in this study is an example), the covariance matrices ***Σ***_***c***_ are not equal throughout all classes. In that case, a QDA [22] is an appropriate solution. The discriminant function *δ*_*c*_(*x*) has a quadratic form, and the decision boundaries between two classes *c*_1_ and *c*_2_ are described by quadratic equations $\left\{x:\delta_{c_{1}}(x)=\delta_{c_{2}}(x)\right\}$. In particular, the discriminant functions are defined by

$$\begin{array}{l} \delta_{c}\left(x^{*}\right)=-\frac{1}{2}\log\left|\Sigma_{c}\right|-\frac{1}{2}{\left(x^{*}-\mu_{c}\right)}^{⊤}\Sigma_{c}^{-1}\left(x^{*}-\mu_{c}\right)\\ \;+\log\pi_{c}, \end{array}$$

and are thus quadratic in *x*^∗^.

#### *Penalized discriminant analysis (PDA)*

A PDA [23,24] is a penalized version of the LDA, which is a sensible extension in the case of a large number of highly correlated covariables such as discretized spectra.

Mathematically, many correlated covariables will result in a singular, i.e. non-invertible covariance matrix ***Σ***, thus making the classification very instable or even infeasible. Hastie et al. [23] replace ***Σ*** by a regularized version, ***Σ*** +*λ* ***Ω***, where ***Ω*** is a roughness-penalizing matrix and *λ* is a smoothing parameter that adjusts the amount of smoothing. The classification then proceeds much like an LDA, but with the penalized covariance matrix.

The choice of ***Ω*** determines the type of penalization used. Common choices are a second-order difference structure that effectively penalizes second derivatives, or a simpler choice of ***Ω*** = *λ* ***I***_*p*_ that results in a penalization similar to ridge regression [24]. We chose the latter matrix, ***Ω*** = *λ* ***I***_*p*_, for regularization.

#### *Classification trees*

Classification trees [12] work by partitioning the covariate space into rectangles and fit simple models (mostly a constant) in each rectangle. The gradual partitioning can be visualized through a *decision tree*, that yields a path along the tree resulting in a constant, i.e. a predicted response (or an above-mentioned simple model) for every new observation. Different methods are available, for example the CART and C4.5 algorithms.

This analysis used CART trees, which are restricted to binary splits at each splitpoint and uses the Gini index as an impurity measure.

To control when the tree stops growing and the model is considered as finished, many hyperparameters can be used. One hyperparameter limits the depth of the tree to some maximum number. Furthermore, one can determine the minimum number of observations that must exist in a node for another split to be attempted, and/or the minimum number of observations in any terminal leaf node. Alternatively, one can set a minimum factor by which the lack of fit has to be decreased in order to execute the next split.

This procedure, however, might stop a tree too early, when e.g. the next split is of little importance, but a subsequent split could lead to a large increase in classification accuracy. Thus, one usually employs a strategy called *pruning*, where first a large tree is grown, and subsequently splits of little importance removed, keeping possible better splits further down the tree. A hyperparameter *α* controls how good a split has to be so that it is kept in the final, pruned tree. In the case of *α* = 0 the solution is the full tree, for *α* = 1 a degenerated tree with zero splits results. Since *α* is the minimum factor by which the lack of fit must be decreased in order for the next split to be executed, it makes sense to only search for *α* in the low end of the possible interval [0,1].

#### *Random forests*

Random forests [25] are an *ensemble method*, constructing a large number of simple classification trees to obtain a majority vote for the predicted response class. They work because simple trees are high-variance yet unbiased methods, and the “averaging” reduces that high variance.

In short, a random forest is constructed by drawing *B* bootstrap samples from the training data and fitting a tree to each of them, randomly selecting a subset of covariates at each splitpoint. The predicted class for new observations is then the majority vote of the *B* fitted trees.

Hyperparameters for random forests include the stopping criteria for each tree, explained above, as well as the total number *B* of trees to grow, and the number of variables that are randomly sampled as candidates at each split. The last parameter is the most influential one regarding the final goodness of fit.

#### *Neural networks*

A neural network for classification [12] is (for the most common case of one hidden layer) a two-stage classification model, consisting of a *hidden layer*, which in turn is made up of *M* so-called hidden units (or neurons) *Z*_*m*_ = *σ*(*α*^⊤^ ***X***), where ***X*** is the covariate matrix. The output layer *g*_*c*_(*T*), where *T* = *β*^⊤^*Z*, is the second step in the two-stage model. The predicted class for an observation is then the class *c* with the largest respective value of *g*_*c*_(*T*). Neural networks are inspired by the workings of neurons in a brain, where they get their name from. The sequential interconnection between the covariates, the neurons and the output layer models the interconnected nature of neurons.

When considering only single layer networks, the main parameter is the number *M* of hidden units in this layer. Furthermore, the activation function *σ*(*v*) can be specified, although it is mostly the sigmoid function, *σ*(*v*) = 1/(1+*e*^-*v*^). Finally, the output function *g*_*c*_(*T*) can be specified, but for classification purposes, it is most commonly the softmax function, $g_{c}(T)=\frac{e^{T_{c}}}{\overset{C}{\underset{\gamma =1}{\sum }}e^{T_{\gamma }}}$.

### Hyperparameter selection

Some classifiers depend on additional hyperparameters which have to be set before the algorithm is run. This section describes which paramters were tuned and how they were chosen.

For a real data set of limited size, the most common method to determine hyperparameters is to execute a cross validation [26], because it most effectively utilizes a limited amount of data. In this simulation setting however, the theoretical possibility to generate data of arbitrary size makes cross validation unnecessary, and so, for each estimation data set, a simple split into one training set (50% of the observations) and one test set (the other 50% of the observations) was carried out. The best hyperparameter was then determined by comparing the total misclassification rate in the test set for a reasonable set of different values for the hyperparameters and choosing the parameter with the lowest respective misclassification rate.

The following hyperparameters were determined: 

• For *k*-nearest-neighbor classification, the parameter *k* was determined by splitting the data into training and test data, computing a fit for all *k*∈{1,…,10}, and choosing for subsequent analysis the value for *k* which resulted in the smallest test set error.

• For random forests, the parameter mtry, i.e. the number of covariates randomly selected for each tree split, was searched for using the procedure tuneRF in the randomForest-package. It compares different values of mtry with their respective out-of-bag errors, but does not try every possible value for mtry, instead it multiplies or divides mtry by a constant factor (2 in this study) at each iteration.

• For classification trees, the complexity parameter *α* was searched for by first growing a large tree with *α*=10^-6^. This tree contains a table of cross-validation errors for several values of *α* larger than 10^-6^, and the optimal value of *α* was extracted from this table and used as the pruning complexity parameter.

• For neural networks, the number of hidden units in the middle layer was searched for from 1 to 4 in the case of spectra, and from 1 to 10 in the case of PC scores. The range for number of units was limited in the case of spectra for computational reasons.

• For PDA, the smoothing parameter *λ* was searched for from 10^*i*^, *i* = -6,…,3.

### Performance evaluation

All algorithms were applied on each of the 1000 estimation data sets. The classifiers were trained on a sample set (50% of each data set, i.e. 400 of the 800 observations), and their performance was subsequently evaluated on an independent test set (the remaining 400 observations).

#### *Distribution estimation of the misclassification rate*

The variation of the misclassification rate was of central interest in this study. The 1000 estimation data sets were generated for that reason. The classification algorithms were applied to the training data of each of these data sets and subsequently evaluated on the test data.

The misclassification rate is the percentage of observations for which a wrong class was predicted. Denoting the covariates (i.e. the spectra) in the test set by ***x***_*i*_, and their respective tissue type by *y*_*i*_, the misclassification rate is defined by

$$\frac{1}{n}\;\#\{i:\;Ŷ(x_{i})\neq y_{i}\}$$

for a test set with *n* observations.

To estimate a distribution of the misclassification rate, for each of the 1000 data sets the classifiers were trained and tested, yielding a sample of misclassification rates. These samples were used to create boxplots of the misclassification rates to allow for a better performance judgment.

#### *Averaged confusion matrices*

To obtain insights into the specific type of mistakes made in the classification, an average of the confusion matrices of the 1000 simulated data sets was computed. Its *C* = 8 rows and *C* = 8 columns are the specific tissue types, and the percentages show what ratio of tissues of type *c* get classified into group *d*. These insights can be helpful to find out what type of tissues are especially difficult to discriminate.

## Results

### Principal component analysis

For all of the the 1000 estimation data sets, the average eigenvalue criterion selected 6 principal components. Therefore, classification of PC scores was always carried out with 6 covariates.

### Averaged confusion matrices

For each of the algorithms, 1000 confusion matrices were generated, one for each of the estimation data sets. Shown are the averages for each investigated algorithm. The confusion matrices show the actual tissue types *y*_*i*_ in the columns, and the classified tissue types $Ŷ(x_{i})$ in the rows. Therefore, (only) the row percentages sum up to 1.

#### *k-nearest-neighbors*

For *k*-nearest neighbors, the PC scores of fat and the salivary gland get confused most often (for 20 and 25 percent of all their respective observations).

Looking at the original spectra, cortical bone and the salivary gland get misclassified most often, both of them into similar tissue types (muscle, nerve, and fat, as well as the respective other tissue type).

Table 2 shows the confusion matrix for this algorithm.

**Table 2:** The average confusion matrices for the KNN algorithm

**PC scores**	**Classified as**							
**True tissue**	**Cancellous Bone**	**Cortical Bone**	**Fat**	**Mucosa**	**Muscle**	**Nerve**	**S. Gland**	**Skin**
Cancellous Bone	*0.94*	0.01	0.00	0.00	0.00	0.00	0.00	0.04
Cortical Bone	0.04	*0.69*	0.05	0.00	0.03	0.06	0.11	0.02
Fat	0.00	0.02	*0.71*	0.01	0.00	0.01	0.25	0.00
Mucosa	0.00	0.00	0.02	*0.86*	0.00	0.09	0.01	0.01
Muscle	0.00	0.00	0.00	0.00	*1.00*	0.00	0.00	0.00
Nerve	0.00	0.03	0.00	0.04	0.00	*0.85*	0.05	0.02
S.Gland	0.00	0.06	0.20	0.00	0.01	0.07	*0.65*	0.01
Skin	0.04	0.00	0.00	0.01	0.00	0.01	0.00	*0.93*
**Spectra**	**Classified as**							
**True tissue**	**Cancellous Bone**	**Cortical Bone**	**Fat**	**Mucosa**	**Muscle**	**Nerve**	**S.Gland**	**Skin**
Cancellous Bone	*0.95*	0.01	0.00	0.00	0.00	0.00	0.00	0.03
Cortical Bone	0.04	*0.71*	0.04	0.00	0.02	0.06	0.11	0.01
Fat	0.00	0.02	*0.78*	0.01	0.00	0.01	0.19	0.00
Mucosa	0.00	0.00	0.02	*0.87*	0.00	0.09	0.02	0.01
Muscle	0.00	0.00	0.00	0.00	*1.00*	0.00	0.00	0.00
Nerve	0.00	0.03	0.00	0.04	0.00	*0.86*	0.05	0.02
S.Gland	0.00	0.07	0.13	0.00	0.01	0.07	*0.72*	0.01
Skin	0.04	0.00	0.00	0.01	0.00	0.01	0.00	*0.94*

#### *LDA*

An LDA also has problems in discriminating fat from the salivary gland, making similar mistakes as a *k*-nearest-neighbors analysis of the PC scores. Also, cortical bone tissue is hard to classify, and is most often misclassified into cancellous bone and muscle tissue. The confusion matrix is shown in Table 3.

**Table 3:** The average confusion matrix for the LDA algorithm, analyzing PC scores

**PC scores**	**Classified as**							
**True tissue**	**Cancellous Bone**	**Cortical Bone**	**Fat**	**Mucosa**	**Muscle**	**Nerve**	**S.Gland**	**Skin**
Cancellous Bone	*0.93*	0.03	0.00	0.00	0.00	0.00	0.00	0.04
Cortical Bone	0.10	*0.74*	0.03	0.00	0.06	0.03	0.03	0.01
Fat	0.00	0.01	*0.83*	0.00	0.00	0.02	0.15	0.00
Mucosa	0.00	0.05	0.00	*0.93*	0.00	0.02	0.00	0.00
Muscle	0.00	0.00	0.00	0.00	*1.00*	0.00	0.00	0.00
Nerve	0.00	0.06	0.01	0.00	0.00	*0.85*	0.07	0.01
S.Gland	0.00	0.01	0.09	0.00	0.00	0.01	*0.89*	0.00
Skin	0.02	0.01	0.00	0.00	0.00	0.00	0.00	*0.97*

#### *Neural networks*

Analyzing the PC scores with neural networks, the common mistake made by other classifiers, i.e. confusing the salivary gland and fat tissues, is also visible here. Cortical bone is again hard to classify, but seems to get misclassified into other tissues with equal frequencies.

When running a neural network on the original spectra, the confusion matrix appears as if it were produced mostly by random guessing. Only cancellous bone was recognized with an increased frequency of 0.40. See Table 4 for the confusion matrix.

**Table 4:** The average confusion matrix for the neural net algorithm

**PC scores**	**Classified as**							
**True tissue**	**Cancellous Bone**	**Cortical Bone**	**Fat**	**Mucosa**	**Muscle**	**Nerve**	**S.Gland**	**Skin**
Cancellous Bone	*0.92*	0.04	0.00	0.00	0.01	0.00	0.00	0.03
Cortical Bone	0.07	*0.78*	0.04	0.01	0.02	0.04	0.03	0.02
Fat	0.00	0.04	*0.81*	0.01	0.00	0.03	0.11	0.00
Mucosa	0.00	0.01	0.01	*0.94*	0.00	0.03	0.00	0.01
Muscle	0.00	0.01	0.00	0.00	*0.98*	0.00	0.00	0.00
Nerve	0.01	0.04	0.02	0.02	0.00	*0.86*	0.04	0.01
S.Gland	0.00	0.02	0.11	0.00	0.00	0.03	*0.82*	0.01
Skin	0.04	0.01	0.00	0.02	0.00	0.01	0.01	*0.91*
**Spectra**	**Classified as**							
**True tissue**	**Cancellous Bone**	**Cortical Bone**	**Fat**	**Mucosa**	**Muscle**	**Nerve**	**S.Gland**	**Skin**
Cancellous Bone	*0.40*	0.06	0.07	0.08	0.14	0.07	0.07	0.11
Cortical Bone	0.13	*0.09*	0.14	0.12	0.16	0.14	0.12	0.09
Fat	0.07	0.07	*0.20*	0.16	0.11	0.17	0.15	0.08
Mucosa	0.08	0.07	0.17	*0.20*	0.12	0.15	0.13	0.09
Muscle	0.15	0.07	0.11	0.11	*0.26*	0.11	0.10	0.09
Nerve	0.07	0.07	0.18	0.15	0.11	*0.17*	0.15	0.08
S.Gland	0.08	0.07	0.17	0.15	0.11	0.16	*0.16*	0.09
Skin	0.21	0.07	0.10	0.12	0.14	0.10	0.10	*0.17*

#### *PDA*

A penalized discriminant analysis made almost no mistakes and correctly classified nearly all of the samples in the test set. A small amount of misclassification occured but is not visible in the confusion matrices due to rounding. This can have a number of reasons, including the nature of the simulation procedure or simply its intrinsic advantage when dealing with functional data. The confusion matrix is shown in Table 5.

**Table 5:** The average confusion matrix for the PDA algorithm, analyzing spectra

**Spectra**	**Classified as**							
**True tissue**	**Cancellous Bone**	**Cortical Bone**	**Fat**	**Mucosa**	**Muscle**	**Nerve**	**S.Gland**	**Skin**
Cancellous Bone	*1.00*	0.00	0.00	0.00	0.00	0.00	0.00	0.00
Cortical Bone	0.00	*1.00*	0.00	0.00	0.00	0.00	0.00	0.00
Fat	0.00	0.00	*1.00*	0.00	0.00	0.00	0.00	0.00
Mucosa	0.00	0.00	0.00	*1.00*	0.00	0.00	0.00	0.00
Muscle	0.00	0.00	0.00	0.00	*1.00*	0.00	0.00	0.00
Nerve	0.00	0.00	0.00	0.00	0.00	*1.00*	0.00	0.00
S.Gland	0.00	0.00	0.00	0.00	0.00	0.00	*1.00*	0.00
Skin	0.00	0.00	0.00	0.00	0.00	0.00	0.00	*1.00*

#### *QDA*

A quadratic discriminant analysis of the PC scores made almost no mistakes, merely misclassifying some cortical bone, fat, nerve, and salivary gland tissues. See Table 6 for the confusion matrix.

**Table 6:** The average confusion matrix for the QDA algorithm, analyzing PC scores

**PC scores**	**Classified as**							
**True tissue**	**Cancellous Bone**	**Cortical Bone**	**Fat**	**Mucosa**	**Muscle**	**Nerve**	**S.Gland**	**Skin**
Cancellous Bone	*1.00*	0.00	0.00	0.00	0.00	0.00	0.00	0.00
Cortical Bone	0.00	*0.98*	0.01	0.00	0.00	0.01	0.00	0.00
Fat	0.00	0.01	*0.98*	0.00	0.00	0.00	0.01	0.00
Mucosa	0.00	0.00	0.00	*1.00*	0.00	0.00	0.00	0.00
Muscle	0.00	0.00	0.00	0.00	*1.00*	0.00	0.00	0.00
Nerve	0.00	0.01	0.00	0.00	0.00	*0.99*	0.00	0.00
S.Gland	0.00	0.00	0.01	0.00	0.00	0.00	*0.98*	0.00
Skin	0.00	0.00	0.00	0.00	0.00	0.00	0.00	*1.00*

#### *Random forests*

Random forests make similar mistakes than other classifiers when analyzing PC scores, most notably the confusion of fat and salivary gland. However, the overall error rate is smaller than for discriminant analyses and *k*-nearest-neighbor classification.

For PC scores, the types of misclassification are similar, but the overall performance suffers noticeably. The resulting confusion matrix is shown in Table 7.

**Table 7:** The average confusion matrix for the random forest algorithm

**PC scores**	**Classified as**							
**True tissue**	**Cancellous Bone**	**Cortical Bone**	**Fat**	**Mucosa**	**Muscle**	**Nerve**	**S.Gland**	**Skin**
Cancellous Bone	*0.97*	0.02	0.00	0.00	0.00	0.01	0.00	0.00
Cortical Bone	0.02	*0.87*	0.02	0.00	0.02	0.02	0.02	0.02
Fat	0.00	0.01	*0.89*	0.00	0.00	0.02	0.08	0.00
Mucosa	0.00	0.01	0.00	*0.97*	0.00	0.01	0.00	0.00
Muscle	0.00	0.00	0.00	0.00	*0.99*	0.00	0.00	0.00
Nerve	0.01	0.03	0.02	0.00	0.00	*0.90*	0.03	0.00
S.Gland	0.00	0.01	0.08	0.00	0.00	0.03	*0.88*	0.00
Skin	0.01	0.01	0.00	0.00	0.00	0.00	0.00	*0.98*
**Spectra**	**Classified as**							
**True tissue**	**Cancellous Bone**	**Cortical Bone**	**Fat**	**Mucosa**	**Muscle**	**Nerve**	**S.Gland**	**Skin**
Cancellous Bone	*0.93*	0.01	0.00	0.00	0.01	0.00	0.00	0.04
Cortical Bone	0.04	*0.70*	0.05	0.01	0.03	0.06	0.08	0.03
Fat	0.00	0.02	*0.79*	0.01	0.00	0.01	0.17	0.00
Mucosa	0.00	0.00	0.02	*0.83*	0.00	0.10	0.02	0.03
Muscle	0.00	0.01	0.00	0.00	*0.98*	0.00	0.00	0.00
Nerve	0.01	0.02	0.00	0.07	0.00	*0.83*	0.03	0.04
S.Gland	0.00	0.09	0.13	0.02	0.01	0.07	*0.67*	0.01
Skin	0.07	0.01	0.00	0.02	0.00	0.02	0.00	*0.89*

#### *Trees*

Trees have a relatively weak performance in this analysis. Analyzing the original spectra with trees show a particularly weak performance. Comparing them with random forests, one can see that the ensemble method does not result in a big improvement in discrimination of cancellous bone and muscle tissue, but improves the discrimination of the six other tissue types. See Table 8 for the confusion matrix of this algorithm.

**Table 8:** The average confusion matrix for the CART (tree) algorithm

**PC scores**	**Classified as**							
**True tissue**	**Cancellous Bone**	**Cortical Bone**	**Fat**	**Mucosa**	**Muscle**	**Nerve**	**S.Gland**	**Skin**
Cancellous Bone	*0.89*	0.05	0.01	0.01	0.02	0.01	0.00	0.01
Cortical Bone	0.05	*0.68*	0.08	0.05	0.03	0.05	0.03	0.04
Fat	0.00	0.06	*0.73*	0.01	0.00	0.05	0.14	0.01
Mucosa	0.03	0.07	0.02	*0.85*	0.00	0.04	0.00	0.00
Muscle	0.01	0.02	0.01	0.01	*0.95*	0.00	0.00	0.00
Nerve	0.04	0.07	0.06	0.03	0.00	*0.74*	0.05	0.00
S.Gland	0.01	0.02	0.17	0.00	0.00	0.07	*0.72*	0.00
Skin	0.05	0.02	0.01	0.01	0.02	0.02	0.02	*0.86*
**Spectra**	**Classified as**							
**True tissue**	**Cancellous Bone**	**Cortical Bone**	**Fat**	**Mucosa**	**Muscle**	**Nerve**	**S.Gland**	**Skin**
Cancellous Bone	*0.87*	0.02	0.00	0.00	0.03	0.00	0.00	0.08
Cortical Bone	0.05	*0.47*	0.08	0.03	0.04	0.09	0.16	0.08
Fat	0.00	0.07	*0.67*	0.01	0.00	0.01	0.23	0.01
Mucosa	0.01	0.01	0.03	*0.67*	0.00	0.18	0.06	0.05
Muscle	0.01	0.01	0.00	0.00	*0.97*	0.00	0.00	0.01
Nerve	0.01	0.03	0.00	0.14	0.00	*0.72*	0.04	0.06
S.Gland	0.01	0.16	0.19	0.05	0.01	0.07	*0.49*	0.03
Skin	0.16	0.04	0.00	0.04	0.00	0.05	0.03	*0.67*

### Misclassification rates

**Figure 3:**
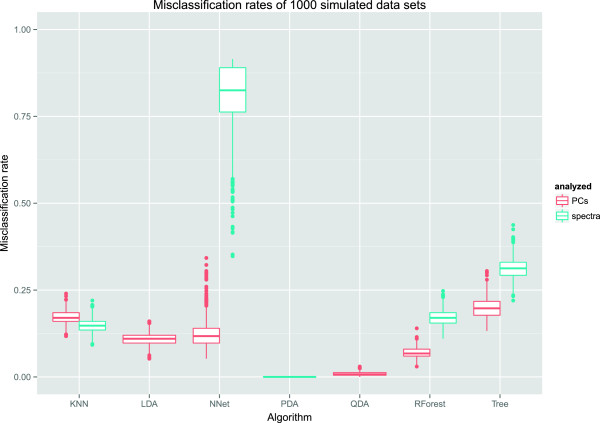
**Misclassification rates of the investigated algorithms for 1000 simulated data sets.** For LDA and QDA, only the PC scores were analyzed, and for PDA, only the original spectra were analyzed. The results suggest that the PDA and QDA algorithms are the most appropriate classifiers for this type of problem.

The 1000 misclassification rates per algorithm were summarized into quantiles. The 0% quantile is equal to the minimum, the 50% quantile is the median, and the 100% quantile is the maximum of each vector. The quantiles are shown in Table 9 and depicted as boxplots in Figure 3.

**Table 9:** Quantiles of the misclassification rates in the 1000 simulated data sets

	**0%**	**25%**	**50%**	**75%**	**100%**
KNN PCs	0.12	0.16	0.17	0.19	0.24
KNN spectra	0.09	0.14	0.15	0.16	0.22
LDA PCs	0.05	0.10	0.11	0.12	0.16
NNet PCs	0.05	0.10	0.12	0.14	0.34
NNet spectra	0.35	0.76	0.82	0.89	0.92
PDA spectra	0.00	0.00	0.00	0.00	0.00
QDA PCs	0.00	0.01	0.01	0.01	0.03
RForest PCs	0.03	0.06	0.07	0.08	0.14
RForest spectra	0.11	0.16	0.17	0.19	0.25
Tree PCs	0.13	0.18	0.20	0.22	0.31
Tree spectra	0.22	0.29	0.31	0.33	0.44

The worst performing algorithms were neural nets with original spectra and the tree algorithms, both for PC scores and original spectra. However, as noted above trees were not tuned because of the many available hyperparameters, and thus might have resulted in a better performance after tuning.

The best performances were achieved by a quadratic discriminant analysis of the PC scores and a penalized discriminant analysis of the original spectra. Both achieve a near-perfect classification with minimal variation throughout the 1000 data sets.

The remaining algorithms had a similar performance, with median error rates roughly between 10 and 20 percent. Random forests showed an improved performance when analyzing PC scores. Taking into consideration that there were eight tissue types, i.e. random guessing would have resulted in a misclassification rate of 7/8, most of the algorithms perform strongly in this study.

### Confirmatory cross validation on the original spectra

The simulation procedure used to generate the data was based on a multivariate normal distribution. Thus, models which impose a normal distribution on the data can be overoptimistic in the classification procedure [12]. This applies to the discriminant analyses, i.e. LDA, QDA and PDA. Because these classifiers assume normal distributions for each of the class densities, the discriminant analyses, and in particular QDA, which allows for different covariance matrices ***Σ***_*c*_ in the classes *c*, could show a reduction in classification performance for real, non-simulated data. One method of avoiding this problem might have been to carry out another simulation procedure, i.e. one that does not rely on a normal distribution.

**Figure 4:**
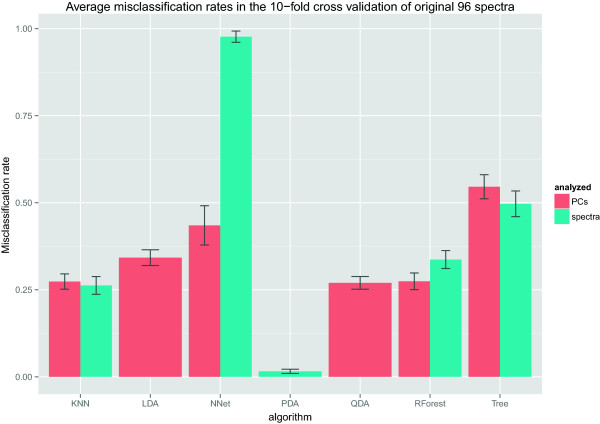
**A 10-fold cross validation of the original data.** When analyzing non-simulated data, LDA and QDA show a weaker performance than in the simulated data sets. PDA also suffers a bit, but still achieves a very high accuracy. The random partitioning of the data into 10 folds was performed 50 times, and the resulting standard deviations over 50 repetitions are shown in the error bars.

To get a picture of the extent of overoptimism of the discriminant analyses, all of the classifiers have been applied to the original (i.e. non-simulated) 96 spectra. Since the original data cannot be arbitrarily augmented through simulation, in this case a cross validation [26] makes sense. Therefore, the data set has been split into ten groups of about equal size (six groups of ten spectra, and four groups of nine spectra). Subsequently, the training and validation procedure was applied ten times for each algorithm, each time using nine of the ten groups as training data and the remaining group as a test data. The resulting misclassification rates for the ten groups were then averaged and are shown in Table 10 and displayed in Figure 4.

**Table 10:** Average misclassification rates in a10-fold cross validation

**Algorithm**	**Misclassification rate**
KNN PCs	0.27
KNN spectra	0.26
LDA PCs	0.34
NNet spectra	0.98
NNet PCs	0.43
PDA spectra	0.02
QDA PCs	0.27
RForest PCs	0.27
RForest spectra	0.34
Tree PCs	0.55
Tree spectra	0.50

The figure shows that the linear and quadratic discriminant analyses now perform similar to random forests and *k*-nearest-neighbors. Untuned trees perform a bit weaker. It is interesting that a penalized discriminant analysis still performs much better than all the other algorithms, and achieves a near-perfect classification accuracy even for real data.

## Discussion

When looking at the classifiers that were applied to both PC scores and original spectra, it seems that reducing the dimensionality, i.e. the amount of information, with a PCA results in a better performance for most algorithms. From the simulation study, a quadratic and a penalized discriminant analysis were near-perfect regarding the test-set error. Furthermore, these two classifiers showed almost no variability when repeating the analysis with 1000 data sets. However, a cross validation on the original data set showed that a QDA exhibits a large amount of overoptimism due to the details of the simulation procedure.

Comparing the results of this study with the previous analyses of Stelzle et al. [2,7], one can see similar results for the LDA of PC scores. Small differences arise, of course, because of the different data preprocessing. A PDA seems to be a much better choice than the previously applied PCA and LDA.

Further research can be targeted at answering several still open questions:

A new sample of original data can be helpful in validating the results of this simulation study. Because the original data come from only twelve animals, the models we computed are probably not robust enough to be employed in a real surgery scenario. However, we believe a comparison study is feasible and insightful even with a smaller sample size, especially with the subsequent validation we performed. Still, it would be insightful to obtain spectra of a larger number of animals, ideally before and after laser ablation, and—barring ethical concerns—of live tissue.

The performance measure considered in this study was a simple misclassification rate, which does not consider the severity of special kinds of misclassification. Further analyses could take that fact into consideration. For example, if a laser scalpel would be disabled near nerve tissue as well as blood vessels, for the final system it would not matter much if a nerve spectrum would be classified as a blood vessel, since the laser is shut down anyway.

Similarly, a binary division of tissues into “critical” and “non-critical” might be considered for further studies. Then, the performance measures can be extended to measure precision and recall, amongst others.

It would be of interest under which circumstances misclassification occurs. If a laser scalpel is approaching nerve tissue during a surgery, an undiscovered measurement of nerve tissue would be less problematic if the following measurement is correctly classified as nerve tissue. However, if the nerve tissue remains undiscovered even throughout repeated measurements, the tissue can be severely damaged before it is noticed. It is therefore interesting to find out if misclassifications occur randomly or show some repeated structure.

The superior performance of PDA, which considers the correlated, functional nature of the covariates, hints that functional approaches may have an advantage in this type of problem. The functional patterns in spectral data carry additional information which can be leveraged by such approaches. It would be interesting for further studies to investigate functional models [3] and compare their performance to the results obtained in this study.

Extending on the previous point, a functional PCA [11], which imposes a smoothness constraint on the eigenvectors (thus making them *eigenfunctions*) can be of interest.

A similar application for these results is the case of tumor detection by spectroscopy. Tumor detection aims to classify the reflectance spectra of tissues into tumor tissue vs. normal tissue. In this case, since there is no surgery going on while the spectra have to be analyzed, time is not of concern. For that reason, other, more timeconsuming methods of spectroscopy can be used. Raman spectroscopy [27] is one of these methods that could provide more accurate results, but takes a greater amount of time to measure the reflectance spectra.

## Conclusion

To conclude, this study confirmed the results of previous studies such as Stelzle et al. [2,7], who have found that classification of diffuse reflectance spectra of different tissue types is a feasible way of improving the safety of laser surgery. As an extension, in this study a QDA and a PDA turned out to be a notable improvement to the previously applied LDA. PDA has shown an equally well performance on a cross-validation on original, i.e. non-simulated data and should be considered for further studies. Additionally, functional data approaches [3] seem like another promising improvement and could be investigated in further studies.

## Competing interests

The authors declare that they have no competing interests.

## Authors’ contributions

AE performed the statistical analyses and wrote the manuscript. WA and MS had the idea for the study and provided methodological guidance throughout the analysis. FS and CK conceived the project, performed the experiments and collected the data. RK contributed to ideas. All authors revised the manuscript and approved the final version.

## Pre-publication history

The pre-publication history for this paper can be accessed here:

http://www.biomedcentral.com/1471-2288/14/91/prepub
